# Comparative analysis of human sperm glycocalyx from different freezability ejaculates by lectin microarray and identification of ABA as sperm freezability biomarker

**DOI:** 10.1186/s12014-018-9195-z

**Published:** 2018-04-30

**Authors:** Ai-jie Xin, Yan-cheng Wu, Hui Lu, Li Cheng, Yi-hua Gu, Hua Diao, Guo-wu Chen, Bin Wu, Zheng Li, Sheng-ce Tao, Xiao-xi Sun, Hui-juan Shi

**Affiliations:** 1 Shanghai Ji Ai Genetics and IVF Institute, Obstetrics and Gynecology Hospital, Fudan University, Shanghai, 200011 China; 2 Key Laboratory of Female Reproductive Endocrine Related Disease, Obstetrics and Gynecology Hospital, Fudan University, Shanghai, 200011 China; 3 China National Population and Family Planning Key Laboratory of Contraceptive Drugs and Devices, SIPPR, Shanghai, 200032 China; 4 Shanghai Key Laboratory of Reproductive Medicine, Shanghai Jiao Tong University School of Medicine, Shanghai, 200025 China; 5 Shanghai Center for Systems Biomedicine, Key Laboratory of Systems Biomedicine (Ministry of Education), Shanghai Jiao Tong University, Shanghai, 200240 China; 6 Department of Allergy, Renji Hospital, Shanghai Jiao Tong University School of Medicine, Shanghai, 200001 China; 7 Department of Andrology and PFD, Center for Men’s Health, Institute of Urology, Urologic Medical Center, Shanghai General Hospital, Shanghai Key Lab of Reproductive Medicine, Shanghai Jiao Tong University, Shanghai, 200080 China

**Keywords:** Cryopreservation, Freezability, Lecin microarray, Human sperm, Glycocalyx

## Abstract

**Background:**

Semen cryopreservation has been widely applied in assisted reproductive technologies and sperm bank, but it causes considerable impairments on sperm quality. It is necessary to find an evaluation indicator for determining the sperm-freezing tolerance.

**Methods:**

The glycocalyx of good freezability ejaculates was compared with poor freezability ejaculates by lectin microarray. The significant different lectins were validated by flow cytometry (FACS). To analyze the relationship between the potential biomarker and the tolerance of sperm to cryopreservation, 60 samples with different recovery rates were collected and detected the lectin-binding intensity by FACS. The receiver operating characteristic (ROC) curve was analyzed to test the capability of the lectin as a potential biomarker for detecting the sperm freezablility.

**Results:**

ABA and DSL were found to develop significant differences between them. Further validation showed that ABA was significantly negative correlated with the sperm recovery rates (*r* = − 0.618, *P* < 0.000) and could be a potential biomarker for predicting sperm freezability (AUC = 0.733 ± 0.067, 95% CI 0.601 − 0.865,* P* < 0.01).

**Conclusion:**

ABA could be a potential biomarker for predicting sperm freezability. It will help to reduce sperm-freezing recovery tests and improve the efficiency of cryopreservation in human sperm bank.

**Electronic supplementary material:**

The online version of this article (10.1186/s12014-018-9195-z) contains supplementary material, which is available to authorized users.

## Background

After decades of development, semen cryopreservation has been widely applied in assisted reproductive technologies (ART) and sperm bank [1, 2]. The technology helps men with azoospermia or severe hereditary disease related with infertility have the opportunity to have children using the donor’s sperm from sperm bank by in vitro fertilization (IVF) or intracytoplasmic sperm injection (ICSI). In addition, for some patients who are about to undergo chemotherapy treatment or other events associated with loss of fertility, sperm cryopreservation can preserve their fertility and make them have their own children later [3, 4]. However, cryopreservation caused dramatic impairments to sperm containing sperm motility, viability, DNA integrity, plasma membrane, and matrix density of mitochondrial, and reduced the sperm ability of penetration of cervical mucus and egg [5–9]. The improvement of the cryopreservation technology and the stability of the recovery rate directly impact on pregnancy rate.

In order to ensure the safe and effective supply of semen, the better quality, higher recovery rate of semen is the working focus of sperm bank. However, the tolerance of sperm to cryopreservation varies in individuals. There are always some semen samples presenting poor ability to resist cryopreservation in clinic. Until now, many studies on human semen have attempted to find biomarkers of freezability. But, some of them showed controversial. Pre-freezed motility has been reported to be correlated with cryosurvival rate [10, 11]. High initial motility and sperm density result in high recovery rate [12]. While other studies reported that the parameters of conventional semen analysis including sperm concentration, motility, WHO morphology and total motile count showed no correlation with the sperm motility recovery rates [13–15]. These suggested that the conventional semen parameters have no sufficient capacity to predict the sperm freezability. In addition, the traditionary method by post-thawed recovery is not only time-consuming, but also labor and reagents wasting, which makes an evaluation indicator for determining the sperm freezing tolerance urgent.

The sperm glycocalyx is a dense carbohydrate layer with 20–60 nm thick, coating on the sperm membrane outmost surface with protein and lipid [16]. It plays an important role in sperm maturation, motility and fertilization [16–18]. During the process of sperm formation, maturation, capacitation and acrosome reaction, the glycoprotein on the sperm surface is largely rearranged. Subtle change in glycocalyx has significant effect on sperm fertility [17, 19–22]. Reports about the cryo-damage of sperm glycocalyx are few due to the technical limitation. Only several papers about the avian sperm carbohydrate changes caused by cryopreservation. And its alterations were associated with the impaired fertility [23, 24]. As a group of natural glycan binders, lectins labeled with different conjugates can detect individual glycans by immunocytochemistry, immunohistochemistry or flow cytome. This is the main method to study the composition of cell glycocalyx. Recently, our lab reported a sensitive and high-through technology-lectin microarray to analyze the sperm glycocalyx [22, 25]. It accelerated the study on sperm glycocalyx.

Therefore, the aim of this study was to find the biomarkers related with human sperm freezability through comparing the glycocalyx between good freezability ejaculates (GFEs) and poor freezability ejaculates (PFEs) by lectin microarray. These will be conducive to optimization of sperm cryopreservation methods, screening of the high quality sperm and improvement of the sperm fertilization.

## Methods

### Sperm collection

All the semen samples in this study were collected in Human Sperm Bank of Renji Hospital, Shanghai Jiao Tong University School of Medicine. The age of the donors ranged from 20 to 35 years. The donors were instructed to collect semen samples through masturbation after 3–5 days sexual abstinence. Semen was harvested in sterile containers. All these samples were evaluated for volume, sperm concentration, total motility, progressive motility (PR), and non-progressive motility (NP) according to the fifth edition of WHO laboratory manual. The samples with normal semen parameters were included; i.e., they presented the normal volume (≥ 2 ml), concentration (≥ 15 × 10^6^/ml) and total motility (≥ 40%).

Liquefied semen samples were divided into two aliquots. One of the aliquots was performed to cryopreservation. The other aliquot was directly fixed for lectin microarray analysis or flow cytometry.

All the donors have given the written informed consent. This research was approved by the Institutional Review Committee of Shanghai Jiao Tong University. All experiments were performed in accordance with the relevant guidelines and regulations.

### Semen cryopreservation and thawing

The semen samples were cryopreserved by direct vapor nitrogen freezing method [26]. The liquefied semen samples were mixed with an equal volume of CryoSperm™ (ORIGIO, USA), followed with incubation at room temperature (RT) for 10 min. Then, the equilibrated samples were transferred to cryovials and placed about 10 cm top from the surface of liquid nitrogen. After incubated for 20 h, the cryovials were preserved in liquid nitrogen at − 196 °C. After 24 h, the cryovials were immediately moved from liquid nitrogen into water bath at 37 °C for 5 min. The sperm total motility was examined to calculate the recovery rate. The recovery rate (%) = Sperm motility after cryopreservation/Sperm motility before cryopreservation × 100%.

### Sperm preparation and lectin microarray analysis

The sperm samples were prepared for lectin microarray analysis as our previously described [22, 25]. The fresh semen and the frozen-thawed semen were centrifuged (500 g × 10 min) for collecting the sperm cells and washed with PBS, followed with fixation with 2% paraformaldehyde containing 0.2% glutaraldehyde for 15 min, and then wash with PBS once, before stored at 4 °C for the subsequent lectin microarray and flow cytometry experiments.

The preparation of lectin microarray was consistent with our previously reported [22, 25, 27]. Simply, 91 lectins with 1 μg/μl concentration were printed in triplicate on OPPolymer Slide H slides (CapitalBio, Beijing, China). Each slide contained 12 blocks with a matrix with 18 × 16 arrangement. Then, stored at 4 °C overnight for the lectins coated on the surface, the slides were ready for the sperm detection.

Lectin microarray was firstly blocked in 10 mM Tris Buffered Saline with 0.5% (v/v) Tween-20 (TBST) for 1 h at RT and then washed once in PBST for 10 min, followed with twice in PBS for 10 min. The fixed sperm labeled with propidine iodide (PI, 20 μg/ml) was adjusted concentration to 5 × 10^6^ spermatozoa in 200 μl PBS with 50 μM CaCl_2_ and 50 μM MnCl_2_ for each block of lectin microarray, and then incubated in a wet box for 1 h at RT in the dark. Each sample was repeated four times in and between slides with a diagonal manner. After the excess and unbound spermatozoa gently removed by submerging and inverting the slides in PBST, the air-dried slides were scanned with a GenePix 4200A (Molecular Devices, Sunnyvale, CA) at 5 μm resolution with the scanning condition set to 532 nm filter and 40% PMT value.

### Validation by flow cytometry

Sixty samples with different recovery rates were collected and fixed. The seminal parameters and recovery rate of the samples were in Additional file 1. 2 × 10^6^ spermatozoa were re-suspended with 90 μl PBS and added 10 μl fluorescein isothiocyanate (FITC)-labeled lectin with the final concentration 100 μg/ml, then incubated for 30 min at 37 °C in the dark. After that the spermatozoa were washed once and re-suspended with 500 μl PBS, to be analyzed in a Facs Calibur Flow cytometer using WinMID2.9 software.

### Statistical analysis

The binding signals of the sperm with lectin microarray were extracted by GenePix pro 6.0. The signal intensity to the local background noise ratio (SNR) was defined as F532 Mean/B532 Mean, and all the spots’ SNRs of lectin microarray were calculated and normalized. Because of each sample performed four blocks repetition and each lectin having three repeats on each block, each sample had 12 SNRs. All the lectin binding signal data of GFEs and PFEs were averaged, respectively. The cut off value of the positive lectin binding was set as SNR ≥ 2.

Data analysis and graphs were conducted by SPSS 20.0 and GraphPad Prism 5 and all the data was described as the mean ± SEM. The significantly different SNRs and the Geo Mean between the GFEs and the PFEs samples were determined by Independent-Samples T Test. The correlation of ABA and recovery rate was analyzed via Pearson Correlations and Linear Regression. ROC analyses were performed with the ABA binding signal intensity plotted against the PFEs. The area under the ROC curves (AUC) were calculated to evaluate the sperm freezability.

## Results

### ABA and DSL being related with sperm freezabiltiy

**Fig. 1:**
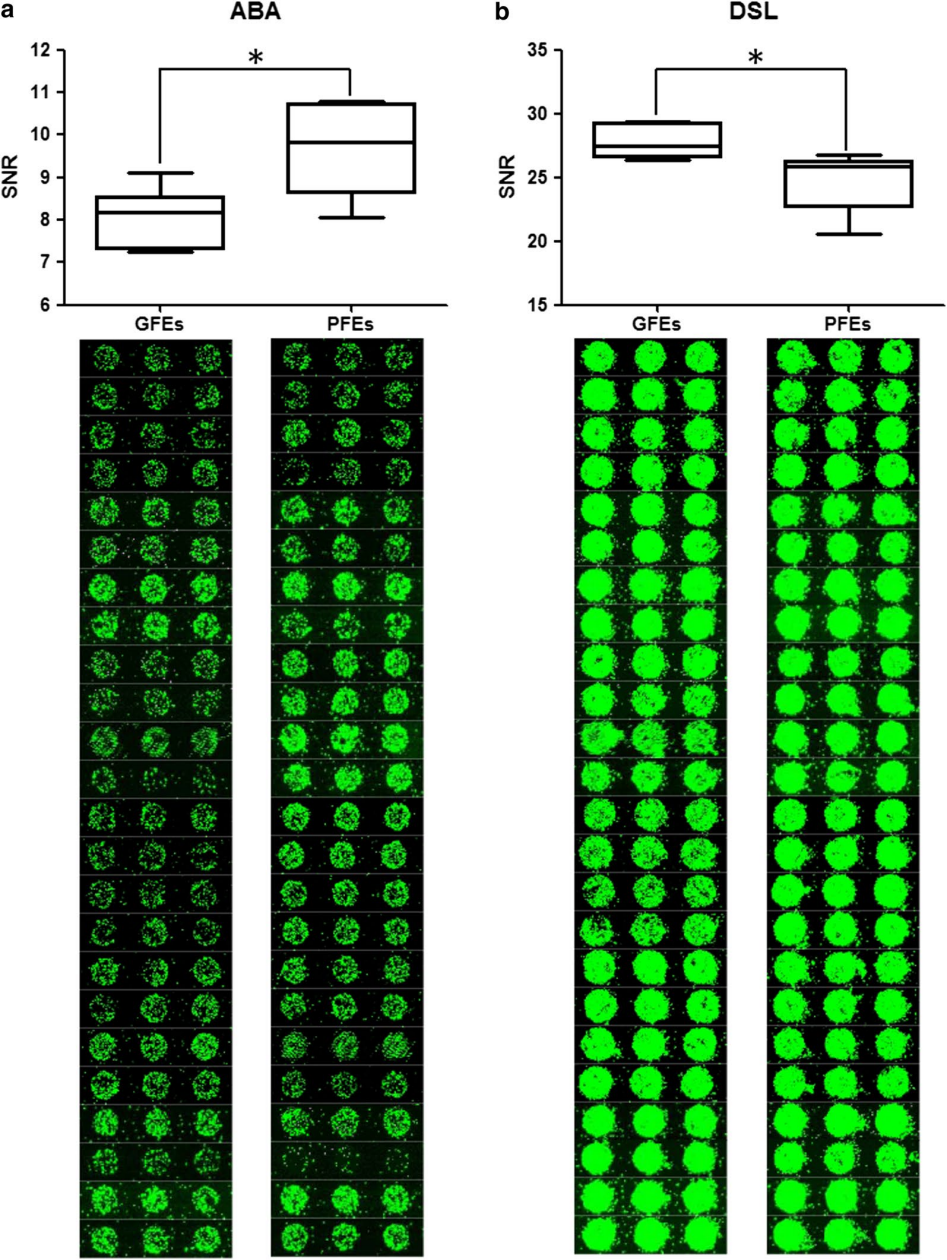
The significantly different lectins between the good freezability ejaculates (GFEs) and the poor freezability ejaculates (PFEs) by lectin microarray. ABA **a** and DSL **b** showing significant difference between GFEs and PFEs, the lower figures presenting the corresponding spots of lectin microarray. *P < 0.05

In order to compare the lectin binding profilings between GFEs and PFEs, we collected 12 semen samples (GFEs, n = 6; PFEs, n = 6) according to the recovery rate. Among them, semen with the recovery rate more than 50% were classified to the GFEs group, while that of less than 30% were classified PFEs. As showed in Table 1, the average age of the enrolled donors was about 26 years, and semen parameters before cryopreservation had no significant difference between the two groups. Obviously, the recovery rate showed statistically different (*P *< 0.000). The GFEs and PFEs samples before cryopreservation were prepared and analyzed by lectin microarray. Through comparation of 91 lectins binding signal intensity between the two groups, *Agaricus bisporusagglutinin* (ABA) and *Datura Stramonium Lectin* (DSL) presented significant difference (Fig. 1). The ABA binding signal intensity of the GFEs showed lower than that of PFEs, while the DSL binding signal intensity was opposite, it was higher in the GFEs.

**Table 1:** The characteristics of the GFEs and the PFEs

	GFEs (n = 6)	PFEs (n = 6)	*P*
Age	26.33 ± 1.82	25.50 ± 1.15	0.707
Semen volume (ml)	3.53 ± 0.62	4.57 ± 0.20	0.145
Sperm concentration (10^6^ per ml)	85.67 ± 12.66	73.67 ± 12.30	0.512
Total motility (PR + NP, %)	57.38 ± 3.92	53.75 ± 3.18	0.489
Recovery rate (%)	58.13 ± 3.00	25.60 ± 1.34	0.000

All values are mean ± SEM

*GFEs* good freezability ejaculates, *PFEs* poor freezability ejaculates, *PR* progressive motility, *NP* non-progressive motility

### Validation of ABA and DSL by FACS

**Fig. 2:**
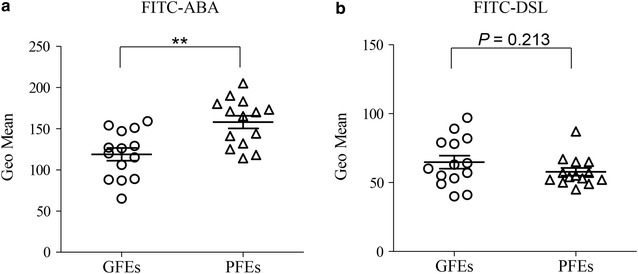
Validation of sperm-lectin binding by flow cytometry. FACS analysis of the good freezability ejaculates (GFEs) and the poor freezability ejaculates (PFEs) labeled with FITC-ABA **a** or FITC-DLS **b**. ABA showing significant difference consistent with the results by lectin microarray; while DLS showing no statistical difference. **P < 0.01

To validate the different lectins binding between GFEs and PFEs, we used fluorescein isothiocyanate (FITC)-labeled ABA and DSL to analyze the binding signal of sperm by flow cytometry (FACS). As indicated in Fig. 2, the fluorescence intensity of ABA in PFEs was significant increased than GEFs, which was totally consistent with the result of lectin microarray. However, the binding signal of DSL demonstrated no significant difference between the two groups. So, ABA was screened for the further experiments.

### ABA being a potential biomarker for detecting the sperm freezability

**Fig. 3:**
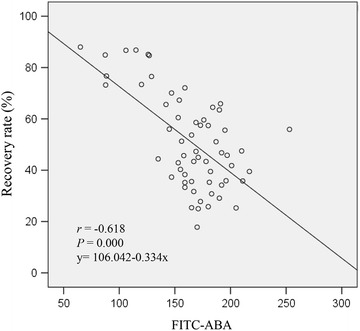
Correlation of ABA and the recovery rate of human sperm. ABA was significantly negative correlated with the sperm recovery rates

To analyze the relationship between ABA and the tolerance of sperm to cryopreservation, sixty samples with different recovery rates were collected and detected the ABA binding intensity by FACS. The Pearson Correlation Coefficients demonstrated the binding intensity with ABA was significantly negative correlated with the sperm recovery rate (*r* = − 0.618, *P *< 0.000). In addition, the linear regression relationship between them is as: y = 106.042 − 0.334x (Fig. 3).

**Fig. 4:**
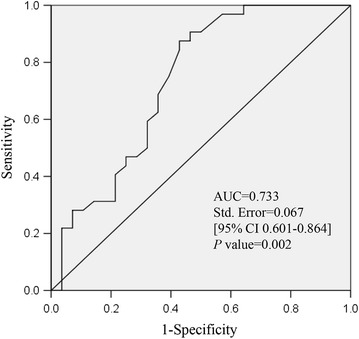
Evaluation of ABA being biomarker for sperm freezability. The good freezability ejaculates as the control group (n = 32) and the poor freezability ejaculates as abnormal group (n = 28). The ROC curves and the corresponding AUCs were calculated by SPSS16.0

Furthermore, to test the capability of ABA as a potential biomarker for detecting the sperm freezability, Receiver Operating Characteristic (ROC) curve was analyzed. The recovery rate of 50% was set as the cut-off value of the tolerance of sperm to cryopreservation. As showed in Fig. 4, the area under the curves (AUC) of ABA was 0.733 ± 0.067 (95% CI 0.601–0.865, *P *< 0.01), which indicated that ABA could serve as a potential biomarker for detecting the sperm freezability. The cut-off value of ABA based on the data was 157 with 57.1% specificity (95% CI 0.372–0.755) and 87.5% sensitivity (95% CI 0.710–0.965).

## Discussion

High quality semen is the core and key of sperm bank. However, there are often some semen samples have better semen parameters before freezing, but poor and unqualified motility after cryopreservation. Prediction of sperm freezability and classification of human semen into GFEs or PFEs before cryopreservation will help to save time, money and labor and facilitate the full use of frozen-thawed human spermatozoa. In this study, by comparing the lectin binding profilings of sperm samples with high recovery rate (classified to GFEs) with that of sperm with low recovery rate (classified to PFEs) through the most comprehensive lectin microarray technology, ABA and DSL showed significant differences, and ABA had the biomarker potential for detecting the sperm freezability.

Sperm glycocalyx, composed of glycoproteins and glycolipids, located on the outer surface of the plasma membrane, protects sperm from the immune surveillance in the uterus and helps maintain sperm survival [28–30]. It is reported that cryopreservation changes the sperm carbohydrates in avian, and that is associated with the impaired fertility [23, 24]. In addition, we found that cryopreservation significantly changed the sperm glycocalyx in human, and the sialic acid, generally located at the terminal of the sugar chain of glycoprotein, was seriously lost [31]. This suggested to some extent that the glycocalyx played an important role on protecting sperm from cryopreservation. Studies of the effects of cryopreservation on spermatozoa mainly focused on proteomics of seminal plasma or sperm membranes in many mammal species [32–36]. To the best of our knowledge, this is the first study that associate the glycocalyx with semen freezabiltiy in human.

It is reported that Kruger strict morphology is significantly correlated with the progressive motility recovery rate (*r* = 0.294, *P *= 0.028) and marginally significant with the relationship between cryosurvival rate (*r* = 0.249, *P *= 0.064) [15]. While we found that the Pearson Correlation Coefficients of ABA and sperm recovery rates (*r* = − 0.618, *P *< 0.000) was more relevant and significant than Kruger morphology. Jiang et al. [37] reported a multivariate model for predicting semen cryopreservation outcomes by three semen parameters, including progressive motility (PR), straight-line velocity (VSL) and average path velocity (VAP), and the AUC of the multivariate model is 0.789. In this study, the AUC of ABA was 0.733. It was illustrated that the one factor of ABA had considerable predictive capacity compared with the multivariate model.

According to the glycosylation site of the peptide chain, glycoproteins possess two type glycans, N-linked glycans and O-linked glycans. N-acetylgalactosamine (GalNAc) is generally added to serine (Ser) or threonine (Thr) residues at the first step in O-glycosylation of proteins by polypeptide N-acetylgalactosaminyl transferase-6 (pp-GalNAc-T6) catalyzation [38, 39], followed by galactose (Gal) and N-Acetylglucosamine (GlcNAc) transferation and added sialic acid (Sia) at the terminal of glycan chains. The lectin ABA specifically recognized the oligosaccharides of O-linked glycosylation (GalNAc-Ser/Thr) which was generally located in the inside of glycocalyx. In this study, the binding signal of ABA was significantly increased in PFEs. The sperm glycocalyx coated on the outmost surface of sperm membrane and played an important role on protecting sperm. The increase of ABA might be due to the imperfect glycocalyx and exposure of the inner oligosaccharides in PFEs. In addition, the previous paper in our lab had been found that the cryo-damaged sperm showed the higher binding intensity to ABA than the fresh sperm [31]. It is suggested that the glycocalyx of PEFs had been impaired when ejaculated, and had no competence to resist cryopreservation.

It is reported that the protein composition has significant difference between GFEs and PFEs, and related with sperm motility and fertility [36, 37]. In this study, the sperm glycocalyx between them also showed significant difference. It is reported that the alteration of spermatozoal glycocalyx is associated with impaired fertility in the fowl [40]. It seems reasonable to hypothesize that sperm freezability is the inherent characteristic of sperm, and it varies in individuals. Furthermore, on the basis of mastering the technique of sperm cryopreservation and the quality control in laboratory, sperm freezability should be considered as one of the evaluation indicators of sperm quality. By comparing the glycocalyx between GFEs and PFEs prior to freezing, ABA will be one of potential biomarkers to predict the sperm freezability. This will help to reduce sperm freezing recovery tests, thereby reducing the workload and semen waste and improve the efficiency of cryopreservation in human sperm bank.

## Additional file

**Additional file 1**. The characteristics of the sixty samples with different recovery rates.
