# Supplementary material for: Comparative analysis of human sperm glycocalyx from different freezability ejaculates by lectin microarray and identification of ABA as sperm freezability biomarker
Source: Clin Proteomics. 2018 Apr 30;15:19. doi: 10.1186/s12014-018-9195-z (PMC5925848; doi:10.1186/s12014-018-9195-z)
Supplement: Supplementary file 1 — Additional file 1. The characteristics of the sixty samples with different recovery rates. [file 12014_2018_9195_MOESM1_ESM.docx]

The characteristics of the sixty samples with different recovery rates.

| Sample | Age | Semen volume (ml) | Sperm concentration  (10^6 / ml) | Motility before cryopreservation (%) | Motility after cryopreservation (%) | Recovery rate (%) |
| --- | --- | --- | --- | --- | --- | --- |
| 1 | 24 | 2.0 | 52 | 76 | 33 | 43 |
| 2 | 28 | 4.0 | 56 | 71 | 25 | 35 |
| 3 | 25 | 3.0 | 48 | 75 | 42 | 56 |
| 4 | 24 | 2.1 | 86 | 67 | 17 | 25 |
| 5 | 30 | 3.0 | 94 | 69 | 32 | 46 |
| 6 | 23 | 3.5 | 66 | 62 | 29 | 47 |
| 7 | 28 | 2.8 | 66 | 64 | 47 | 73 |
| 8 | 21 | 5.0 | 102 | 67 | 47 | 70 |
| 9 | 30 | 2.3 | 92 | 64 | 42 | 66 |
| 10 | 28 | 3.0 | 66 | 76 | 66 | 87 |
| 11 | 30 | 3.2 | 65 | 75 | 28 | 37 |
| 12 | 23 | 2.6 | 98 | 73 | 42 | 58 |
| 13 | 32 | 3.0 | 77 | 71 | 52 | 73 |
| 14 | 24 | 4.3 | 67 | 78 | 40 | 51 |
| 15 | 26 | 3.5 | 77 | 55 | 37 | 67 |
| 16 | 28 | 3.8 | 111 | 67 | 27 | 40 |
| 17 | 21 | 4.0 | 105 | 67 | 24 | 36 |
| 18 | 29 | 3.0 | 87 | 67 | 36 | 54 |
| 19 | 33 | 2.5 | 88 | 62 | 40 | 65 |
| 20 | 21 | 2.2 | 70 | 74 | 35 | 47 |
| 21 | 27 | 3.6 | 113 | 56 | 24 | 43 |
| 22 | 31 | 2.2 | 52 | 70 | 32 | 46 |
| 23 | 29 | 4.5 | 86 | 53 | 23 | 43 |
| 24 | 20 | 3.2 | 71 | 60 | 27 | 45 |
| 25 | 20 | 2.5 | 120 | 60 | 23 | 38 |
| 26 | 22 | 3.6 | 67 | 55 | 16 | 29 |
| 27 | 25 | 3.2 | 105 | 68 | 39 | 57 |
| 28 | 20 | 3.0 | 92 | 58 | 34 | 59 |
| 29 | 27 | 3.4 | 62 | 47 | 24 | 51 |
| 30 | 24 | 2.1 | 116 | 59 | 33 | 56 |
| 31 | 30 | 5.0 | 40 | 38 | 10 | 26 |
| 32 | 23 | 4.6 | 51 | 67 | 17 | 25 |
| 33 | 26 | 4.8 | 60 | 35 | 11 | 32 |
| 34 | 26 | 4.8 | 41 | 49 | 17 | 34 |
| 35 | 25 | 2.0 | 86 | 58 | 24 | 42 |
| 36 | 33 | 6.5 | 106 | 45 | 33 | 72 |
| 37 | 27 | 6.4 | 39 | 63 | 22 | 36 |
| 38 | 26 | 4.6 | 98 | 42 | 13 | 31 |
| 39 | 26 | 5.0 | 71 | 46 | 12 | 25 |
| 40 | 23 | 3.5 | 114 | 29 | 11 | 39 |
| 41 | 28 | 2.8 | 67 | 61 | 24 | 39 |
| 42 | 25 | 3.0 | 66 | 41 | 23 | 56 |
| 43 | 28 | 5.0 | 67 | 37 | 16 | 44 |
| 44 | 23 | 3.5 | 91 | 42 | 20 | 48 |
| 45 | 23 | 3.0 | 65 | 40 | 11 | 28 |
| 46 | 25 | 4.0 | 80 | 26 | 9 | 36 |
| 47 | 30 | 3.5 | 60 | 36 | 21 | 60 |
| 48 | 24 | 5.0 | 47 | 47 | 8 | 18 |
| 49 | 22 | 2.5 | 80 | 54 | 35 | 66 |
| 50 | 22 | 4.0 | 100 | 85 | 72 | 85 |
| 51 | 22 | 4.5 | 117 | 30 | 23 | 77 |
| 52 | 22 | 4.0 | 113 | 85 | 54 | 64 |
| 53 | 24 | 2.4 | 65 | 34 | 26 | 76 |
| 54 | 22 | 2.6 | 40 | 90 | 30 | 33 |
| 55 | 23 | 3.6 | 55 | 68 | 24 | 35 |
| 56 | 30 | 3.2 | 102 | 75 | 66 | 88 |
| 57 | 27 | 3.3 | 45 | 53 | 45 | 85 |
| 58 | 31 | 3.2 | 60 | 45 | 39 | 87 |
| 59 | 27 | 5.0 | 42 | 86 | 52 | 60 |
| 60 | 30 | 3.5 | 112 | 87 | 74 | 85 |
